# Quantitative mapping of acute and chronic PCL pathology with 3 T MRI: a prospectively enrolled patient cohort

**DOI:** 10.1186/s40634-019-0188-2

**Published:** 2019-05-28

**Authors:** Katharine J. Wilson, Jurgen Fripp, Carly A. Lockard, Richard C. Shin, Craig Engstrom, Charles P. Ho, Robert F. LaPrade

**Affiliations:** 1 Steadman Philippon Research Institute, Vail, CO USA; 2 The Australian e-Health Research Centre, CSIRO Health and Biosecurity, Level 5 - UQ Health Sciences Building 901/16, Royal Brisbane and Women’s Hospital, Herston, QLD 4029 Australia; 3 School of Human Movement and Nutrition Sciences, Human Movement Studies Building, University of Queensland, St Lucia, QLD 4067 Australia; 4 The Steadman Clinic, Vail, CO USA

**Keywords:** Posterior cruciate ligament, Magnetic resonance imaging, T2 mapping, Knee

## Abstract

**Background:**

The diagnosis of incomplete acute and chronic posterior cruciate ligament (PCL) tears can be challenging with conventional magnetic resonance (MR) imaging, particularly for injuries in which the ligament appears continuous as occurs with chronic PCL tears that have scarred in continuity. Quantitative mapping from MR imaging may provide additional useful diagnostic information in these cases. The purpose of this study was to assess the feasibility of quantifying transverse relaxation time (T2) mapping values at 3 Tesla (T) in a prospectively enrolled patient cohort with chronic PCL tears.

**Methods:**

Twelve subjects with acute or chronic functionally torn PCL, confirmed on clinical exam and posterior knee stress radiographs (with 8 mm or more of increased posterior tibial translation), were enrolled prospectively over a span of 4 years (age: 28–52 years, injury occurred 2 weeks to 15 years prior). Unilateral knee MR images were acquired at 3 T, including a multi-echo spin-echo T2 mapping scan in the sagittal plane. For the six subjects with a continuous PCL on MR imaging the PCL was manually segmented and divided into proximal, mid and distal thirds. Summary statistics for T2 values in each third of the ligament were compiled.

**Results:**

Across the six patient subjects with a continuous ligament, the mean T2 for the entire PCL was 36 ± 9 ms, with the highest T2 values found in the proximal third (proximal: 41 ms, mid 30 ms, distal 37 ms). The T2 values for the entire PCL and for the proximal third subregion were higher than those recently published for asymptomatic volunteers (entire posterior cruciate ligament: 31 ± 5 ms, proximal: 30 ms, mid: 29 ms, distal: 37 ms) with similar methodology.

**Conclusion:**

Mean T2 values were quantified for acute and chronic PCL tears in this prospectively enrolled patient cohort and were higher than those reported for asymptomatic volunteers. This novel approach of using quantitative mapping to highlight injured areas of the posterior cruciate ligament has potential to provide additional diagnostic information in the challenging case of a suspected posterior cruciate ligament tear which appears continuous, including chronic tears that have scarred in continuity and may appear intact on conventional magnetic resonance imaging.

## Background

The posterior cruciate ligament (PCL) is the primary restraint to posterior tibial translation and a secondary restraint to rotation of the knee (LaPrade et al., 2015). Injuries to the PCL are commonly caused by a direct forceful blow to the anterior tibia while the knee is flexed, known as a dashboard type injury, or by less common mechanisms including knee hyperflexion or hyperextension (Servant et al., 2004). Injuries to the PCL often occur concurrently with injury to other knee ligaments and regions, including the anterior cruciate ligament (ACL), medial collateral ligament, and posterolateral corner (LaPrade et al., 2015; Spiridonov et al., 2011). The clinical diagnosis of a PCL injury can be challenging with conventional magnetic resonance (MR) imaging for diagnosing both acute and chronic PCL injuries (DePhillipo et al., 2018; Orakzai et al., 2010; Servant et al., 2004; Tewes et al., 1997). When a PCL tear is suspected, radiologists will commonly examine the patient’s MR images for secondary signs such as PCL thickening, increased signal or elongation (Cho et al., 2001; Draghi et al., 2017; Geeslin et al., 2017; Rodriguez et al., 2008). However, several authors have reported that the appearance of the PCL on conventional MR imaging is not reliable in predicting its functional integrity or degenerative changes (Akisue, Kurosaka, Yoshiya, Kuroda, and Mizuno, 2011; McMonagle et al., 2013; Orakzai et al., 2010; Rodriguez et al., 2008). Particularly for chronic PCL injuries, the ligament may scar in continuity and produce an intact-appearing yet insufficient/lax ligament, complicating diagnosis with conventional MR imaging and resulting in poor sensitivity for chronic PCL tears (DePhillipo et al., 2018; Servant et al., 2004). This outcome has also been identified for acute PCL injuries, with authors reporting a high incidence of cases in which the PCL appeared continuous as a single structure on conventional MR imaging following an acute tear (Akisue, Kurosaka, Yoshiya, Kuroda, and Mizuno, 2011; Rodriguez et al., 2008).

Quantitative mapping may provide additional useful information in the clinical diagnosis of cases in which the injured PCL appears continuous on MR imaging. Quantitative in vivo mapping, particularly transverse relaxation time (T2 and T2*) mapping biomarkers, has been used to study soft tissue degeneration and these biomarker values have been correlated with parameters such as water and collagen content, fiber orientation and tissue stiffness (Fragonas et al., 1998; Lusse et al., 2000; Ross et al., 2013). For instance, T2 mapping in the knee has demonstrated sensitivity to degenerative changes in articular cartilage and the menisci (Chou et al., 2009; Liess et al., 2002; Wang et al., 2016). Quantitative mapping of knee ligament and tendon tissues has been limited, but may provide a surrogate measure for the mechanical characteristics such as strength or integrity of the tissue. Biercevicz et al., showed that T2*-weighted MR images can be used to predict the structural properties of the healing ACL in a porcine model, as well as in the PCL after ACL transection surgery in an ovine model (Biercevicz et al., 2013; Biercevicz et al., 2014). It was demonstrated in ex vivo ligament specimens that the T2* values increased with tissue degeneration (Biercevicz et al., 2013).

There is currently limited reporting of quantitative mapping of in vivo ligament tissue. Normative T2 values and natural variation within the PCL have been established previously in asymptomatic volunteers (Wilson et al., 2016). This previous work demonstrated that there was natural variation within the asymptomatic PCL, with higher T2 values in the proximal region of the PCL (Wilson et al., 2016). An analysis of the pathological PCL using T2 mapping is necessary to determine its clinical usefulness and potential utility as a surrogate measure of tissue degeneration. Therefore, this study was undertaken to quantify T2 mapping values in a prospectively enrolled patient cohort of acute and chronically injured continuous-appearing PCLs with 3 Tesla (T) MR imaging. We hypothesized that these injured PCLs would exhibit regional variation with increased T2 values in the region of suspected injury, and would exhibit higher T2 mapping values than those seen in the literature for asymptomatic subjects.

## Methods

This study was approved by the institutional review board of the Vail Valley Medical Center. Twelve patients with acute or chronic PCL tear were enrolled prospectively in a consecutive series over the span of 4 years in the current study (age: 28–52 years, 10 M/2 F, 7 right/5 left, injury occurred 2 weeks to 15 years prior to imaging). This study included one patient with an acute injury (less than 6 weeks from ligament injury (Xu et al., 2018)) and five patients with chronic injuries (greater than 6 weeks from ligament injury). All patients had a clinical examination and bilateral posterior knee stress radiographs which had a minimum of 8 mm of increased posterior tibial translation compared to the uninjured contralateral knee (Jackman et al., 2008) documenting a PCL tear. Unilateral knee images were acquired on a 3 T MR imaging scanner (Verio, Siemens), including a standard clinical exam and a multi-echo spin-echo T2 mapping scan (sagittal plane; repetition time (TR)/echo time (TE): 2000/10.7–74.9 ms; resolution: 0.6 × 0.6 mm; slice thickness: 2.0 mm; slice gap: 2.4 mm; field of view (FOV): 80 mm; acquisition time (TA): 4 min 44 s). The standard MR imaging exam included: i) a proton-density (PD) fat-suppressed (FS) turbo spin-echo (TSE) sequence (TR/TE: 5300/40 ms; interpolated resolution: 0.19 × 0.19 mm; slice thickness, 3.0 mm; FOV: 120 mm; TA: 2 min 50 s) and ii) a PD TSE sequence (TR/TE: 2500/32 ms; interpolated resolution: 0.16 × 0.16 mm; slice thickness: 3.0 mm; FOV: 125 mm; TA: 3 min 19 s). The injured knee was imaged using a 15-channel multi element phased-array knee coil (Quality Electrodynamics).

**Fig. 1:**
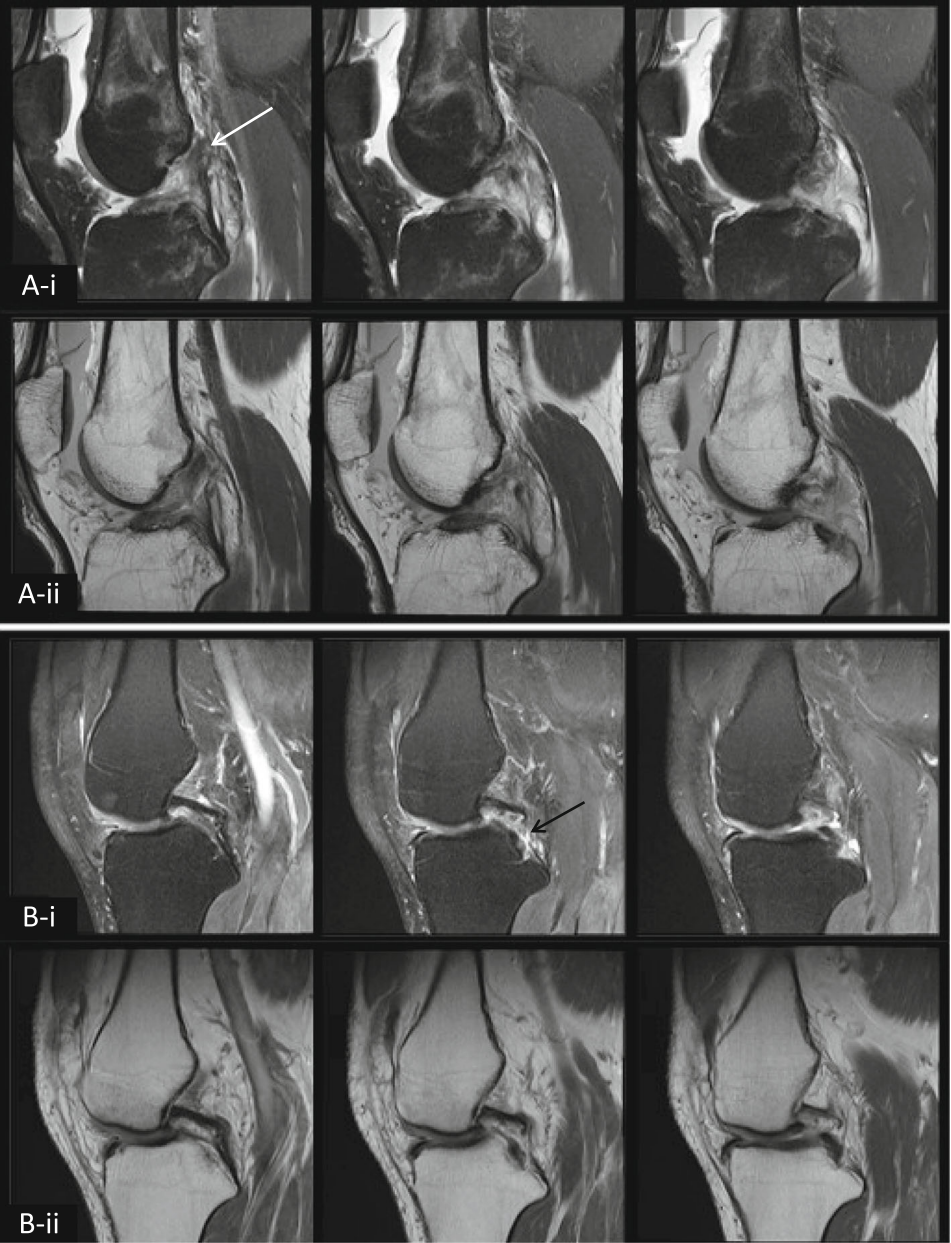
Example images from two subjects with complete PCL tears. Subject A had a chronic injury in which the PCL was detached from the femoral attachment (white arrow). Subject B had a chronic injury in which the PCL was partially torn at its tibial attachment (black arrow). Images shown are from three consecutive slices of i) a PD FS TSE sequence (sagittal plane; TR/TE: 5300/40 ms; interpolated resolution: 0.19 × 0.19 mm; slice thickness: 3.0 mm; FOV: 120 mm; TA: 2 min 50 s) and ii) a PD TSE sequence (sagittal plane; TR/TE: 2500/32 ms; interpolated resolution: 0.16 × 0.16 mm; slice thickness: 3.0 mm; FOV: 125 mm; TA: 3 min 19 s)

Subjects whose PCL appeared fragmented and discontinuous on conventional MR imaging were removed from the T2 mapping analysis, because the fragmented PCL does not represent the same diagnostic dilemma as the scarred-in-continuity PCL, making the application of T2 mapping unnecessary. In addition, the fragmented tissue could not be confidently differentiated from the surrounding tissues, hindering selection/segmentation of PCL tissue. Example images of subjects with complete discontinuous tears of the PCL, which were not included in this T2 mapping analysis, can be seen in Fig. 1.

The remaining 6 subjects, who had continuous-appearing PCLs, were analyzed. Subject demographics and clinical MR imaging information for these 6 subjects is listed in Table 1. For these 6 subjects the PCL was manually segmented in imaging software (Mimics v14, Materialise) by a musculoskeletal radiologist, and automatically divided into proximal, mid and distal thirds by length using custom software (MILXview, CSIRO) as described previously in a published study of an asymptomatic PCL cohort (Wilson et al., 2016). T2 values were calculated for the entire PCL as well as in each region, but were limited to the range of 1 to 250 ms to exclude outliers such as synovial fluid that may have been inadvertently included because of partial volume averaging about the PCL margins.

**Table 1:** Subject demographics, clinical MR imaging information and T2 mapping values for patients with PCL insufficiency showing a continuous ligament on MR images

Subject	Age	Side	Sex	Time from injury to MR imaging	Increase in posterior tibial translation on PCL stress radiographs	Entire PCL T2 (ms)	Proximal T2 (ms)	Mid T2 (ms)	Distal T2 (ms)
1	52	right	F	4 years	8.3 mm	32	31	31	33
2	28	right	M	15 years, re-injured 8 months pre-scan	8.1 mm	46	67	26 +	48
3	33	right	M	9 years, re-injured 5 months pre-scan	8.0 mm	34	38 +	23	36
4	31	left	M	2 years	10.3 mm	23	23	28 +	22
5	29	right	M	2 weeks	13.2 mm	47	53 +	48	44
6	37	right	M	3 months	12.0 mm	34	36 +	22	36
					Mean T2 ± standard deviation:	36 ± 9	41 ± 16	30 ± 9	37 ± 9

Regions of the PCL that were suspected clinically of scarring/injury on conventional MR imaging are marked with a ‘+’. No region was specified in the medical record for subject 1

Summary statistics including the mean and standard deviation for T2 values in each third of the ligament were compiled for further analysis. Due to the varied injury locations and small number of subjects in this case series it was not feasible to test for statistically significant differences between regions or between this PCL injury patient cohort and the T2 mapping values for asymptomatic uninjured PCL subjects in the literature.

## Results

**Fig. 2:**
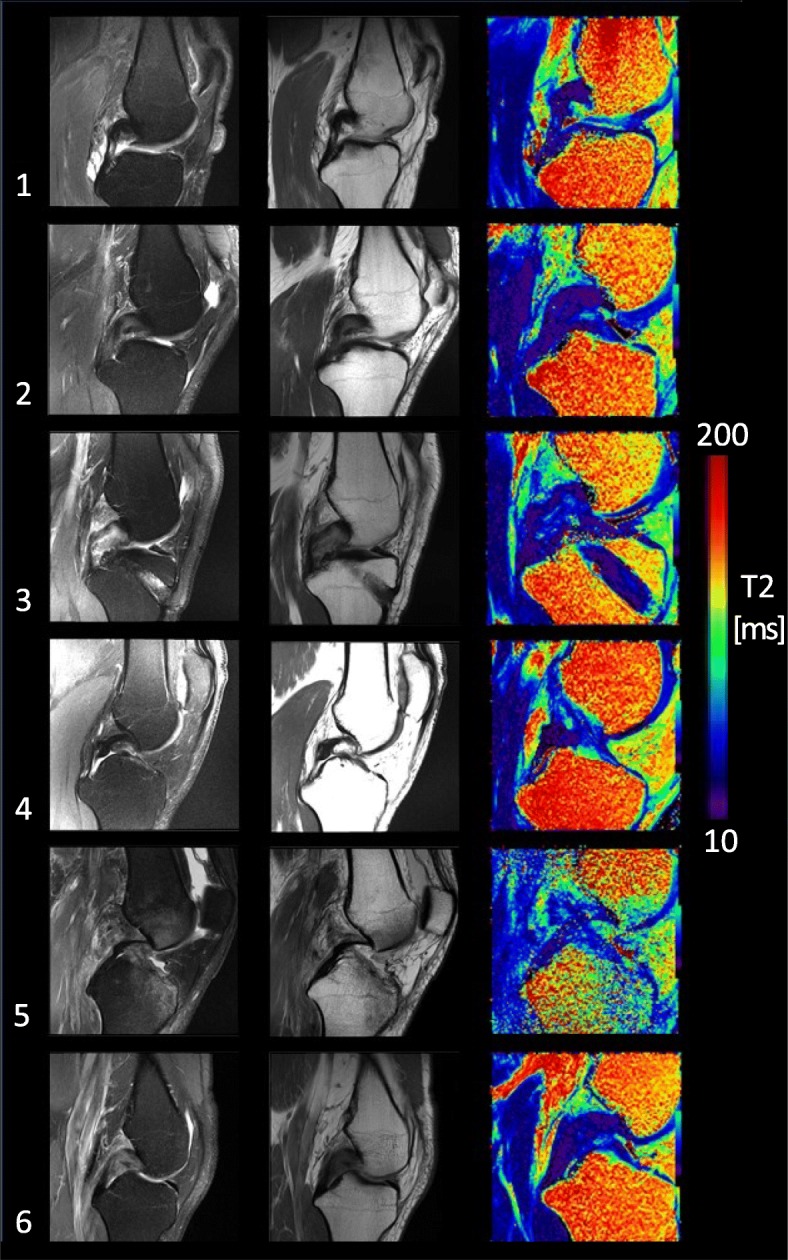
Example MR images in the six individual patients with PCL insufficiency showing a continuous ligament. Images show the closest sagittal slice through the middle of the injured PCL for each of the following sequences: PD FS TSE, PD TSE and color-mapped T2 mapping image

T2 mapping results and MR imaging findings for the 6 subjects with a continuous PCL are summarized in Table 1, with example images shown in Fig. 2. The mean T2 for the entire PCL was 36 ± 9 ms across the 6 subjects. The mean T2 in the proximal region was 41 ± 16 ms, mid-region was 30 ± 9 ms, and distal region was 37 ± 9 ms. The regional mean T2 values in the PCL patients were higher than previously reported for the uninjured PCL in asymptomatic volunteers using the same T2 mapping protocol in the entire PCL and the proximal subregion (entire PCL: 31 ± 5 ms, proximal: 30 ± 7 ms, mid: 29 ± 7 ms, distal: 37 ± 9 ms) (Wilson et al., 2016).

## Discussion

The most important finding of this initial study was that it demonstrated the feasibility of using T2 mapping of the continuous-appearing and functionally torn PCL across a variety of different patients and injuries in a clinical setting with 3 T MR imaging. Increased T2 values have been correlated to increased water content and alterations to the collagen orientation (Liess et al., 2002; Ross et al., 2013), an indication of degenerative changes within the injured PCL. While studies on a larger cohort are needed for further evaluation, the present results demonstrate the potential for T2 mapping to be a useful biomarker in the diagnosis and localization of a chronic PCL tear with ligament continuity.

**Fig. 3:**
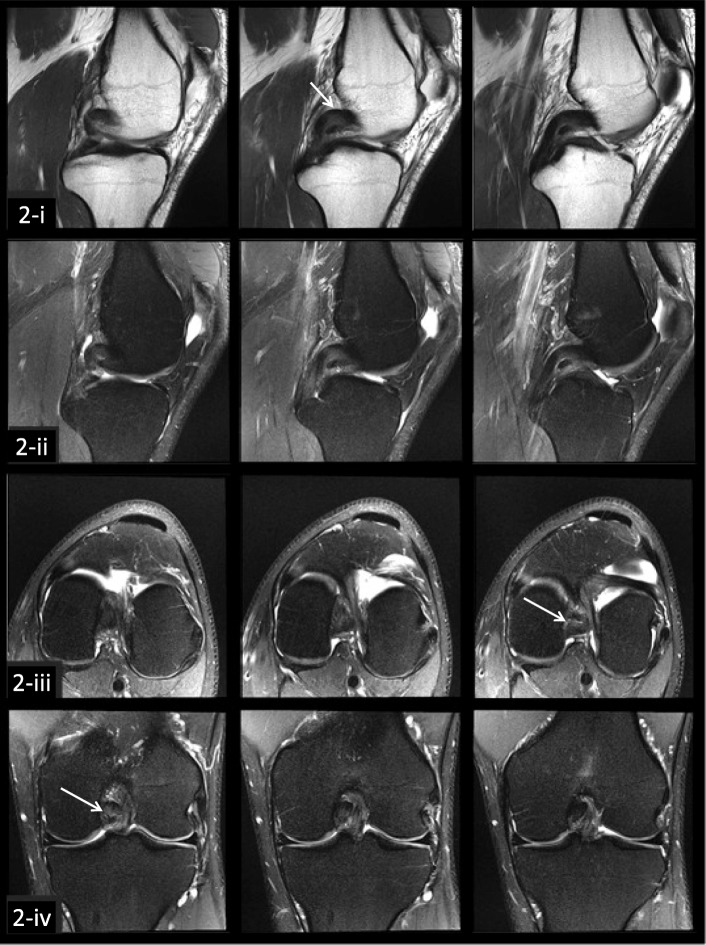
Example images of subject 2 showing three consecutive slices of the PCL from four MR imaging sequences, including i) PD TSE sagittal, ii) PD FS TSE sagittal, iii) PD FS TSE axial, iv) PD FS TSE coronal. This subject was a 28-year old male with a chronic PCL injury from a fall. On original imaging evaluation the radiologist noted minimal focal increased signal within the mid PCL substance likely indicative of mild scarring and/or degeneration. However, a secondary review of the images identified some irregularity in the proximal region of the PCL (white arrows)

The clinical usefulness of quantitative mapping of the PCL was evidenced by the case of subject 2, in which the highest mean T2 value (proximal PCL) did not correspond with the initial clinically identified region in the subject’s medical record (mid-length PCL). However, a second review of the conventional MR images revealed that the proximal region of subject 2 did indeed appear to have scarring or irregularity, (Fig. 3) suggesting that T2 mapping could aid in identifying outliers or challenging cases. Future research examining correlation between injury location on MR imaging acquired in the acute injury period and subsequent T2 mapping value subregion characteristics in the chronic phase may provide a clearer picture of the relationship between subregion T2 distribution and initial PCL injury location.

Injury of the PCL presents a challenge in diagnosis, including acutely injured and especially for chronic PCL tears that appear continuous on MR imaging, due to the variability in tissue disruption patterns. A healthy intact PCL appears as a well-defined continuous band of low signal intensity on MR imaging (Rodriguez et al., 2008; Wilson et al., 2016). In a complete tear the ligament fibers are often discontinuous on MR images, with the fluid signal completely traversing the fibers. In a partial tear, the PCL is often thickened with an increased signal intensity (Rodriguez et al., 2008). However, this is not always the case and more diagnostically challenging injuries of the PCL can include elongation of the ligament which appears as a continuous band on MR imaging but is actually functionally deficient (Servant et al., 2004). These findings present a compelling argument for a quantitative method to aid in clinical diagnosis, particularly for cases where the PCL appears structurally normal on MR images following a knee injury. Including T2 mapping in the clinical MR imaging protocol offers an avenue for additional quantitative information for highlighting injury to the PCL which may not be evident on conventional MR imaging.

In the 6 subjects with a discontinuous tear, the PCL appeared too excessively fragmented on MR imaging to perform accurate selection/segmentation of PCL tissue and T2 mapping analysis. In these subjects, not only were the ligaments difficult to confidently identify and segment for analysis, there was much more substantial risk of partial volume averaging effects in the displaced and indistinct PCL tissue with surrounding synovium and fat and edema/hemorrhage. These factors precluded accurate segmentation and T2 mapping analyses and therefore these cases were excluded from this part of the study. Further, complete discontinuous tear injuries of the PCL are more easily diagnosed with conventional MR imaging, so the clinical need for quantitative mapping is less clear for these cases.

We recognize some limitations to this study. The number of patients was small. However, this initial T2 mapping analysis cohort of 6 patients with an injured but continuous PCL demonstrated the feasibility of using quantitative mapping on a variety of PCL injuries, although a greater number of subjects is required to determine clinical T2 values correlations. Additional data for acute and chronic cases would be of interest to assess how T2 values and spatial distributions change with time from injury.

## Conclusions

In this MR study, T2 values of the PCL were quantified in acute and chronic injuries in a prospectively enrolled cohort of six subjects with injured but continuous-appearing PCLs. This novel approach used quantitative mapping to identify injured areas of the PCL and may provide useful additional information to aid clinical diagnosis. Potentially, T2 mapping offers added benefits for the clinical diagnosis of acute and chronic PCL injuries, particularly when the PCL appears continuous on conventional MR imaging.

## Data Availability

The datasets used and/or analysed during the current study are available from the corresponding author on reasonable request.

## Abbreviations

ACL: Anterior cruciate ligament
FOV: Field of view
FS: Fat-suppressed
mm: Millimeters
MR: Magnetic resonance
ms: Milliseconds
PCL: Posterior cruciate ligament
PD: Proton-density
T: Tesla
T2: Transverse relaxation time (true)
T2*: Transverse relaxation time (effective)
TA: Acquisition time
TSE: Turbo spin echo
